# Quantitative aspects of the E2 receptor assay for human breast tumour cytosol using dextran-coated charcoal.

**DOI:** 10.1038/bjc.1984.206

**Published:** 1984-10

**Authors:** D. W. Wilson, G. Richards, R. I. Nicholson, K. Griffiths

## Abstract

Misclassification of the oestrogen status of a human breast tumour cytosol, arising from different sources and magnitudes of error in the dextran-coated charcoal (DCC) method, have been investigated using both practical and computer simulated data analysed by Scatchard and Mass Action models. The minimum detectable receptor site concentration, relative or absolute numerical bias and imprecision which are complex and integral functions of misclassification, have been calculated from practical data and for a range of experimental conditions likely to be encountered in practice. The Mass Action model was found to be superior and the computer program, designed to investigate the effects of methodological errors on quantitative aspects of the assay, may be a useful aid for analytical design and internal quality control of the receptor assay.


*Br. J. Cancer* (1984), **50**, 493–499

### Quantitative aspects of the $E_2$ receptor assay for human breast tumour cytosol using dextran-coated charcoal

D.W. Wilson, G. Richards, R.I. Nicholson and K. Griffiths

*Tenovus Institute for Cancer Research, Welsh National School of Medicine, Heath Park, Cardiff CF4 4XX, UK.*

**Summary** Misclassification of the oestrogen status of a human breast tumour cytosol, arising from different sources and magnitudes of error in the dextran-coated charcoal (DCC) method, have been investigated using both practical and computer simulated data analysed by Scatchard and Mass Action models. The minimum detectable receptor site concentration, relative or absolute numerical bias and imprecision which are complex and integral functions of misclassification, have been calculated from practical data and for a range of experimental conditions likely to be encountered in practice. The Mass Action model was found to be superior and the computer program, designed to investigate the effects of methodological errors on quantitative aspects of the assay, may be a useful aid for analytical design and internal quality control of the receptor assay.

There is now considerable evidence to indicate that the management of patients with carcinoma of the breast can be improved by the use of oestradiol-17$\beta$ receptor (ER) assays (McGuire *et al.*, 1975). In patients with advanced disease, there is a good correlation between the receptor status of metastatic tissue and response to endocrine therapy (McGuire *et al.*, 1978). Furthermore, not only has a good relationship been observed between the receptor status of the primary tumour and subsequent response of the patient when the disease recurs (Jensen *et al.*, 1976; Nicholson *et al.*, 1981) but the receptor status, in association with various clinical parameters, such as tumour grade and nodal involvement, may be of particular value for the selection of high risk patients for adjuvant therapy following mastectomy (Haybittle *et al.*, 1982). A recent gathering of experts (Consensus Meeting, 1980), has, however, confirmed the effectiveness of the dextran-coated charcoal (DCC) receptor assay for the determination of receptor levels and it now forms the basis on which prognostic investigations are currently being established.

This report evaluates the effect of certain practical inefficiencies in this methodology, such as the presence of non-specific binding which can affect quantitation (Wilson *et al.*, 1971; Chamness & McGuire, 1975). By using the appropriate model for curve-fitting of binding data, the study provided estimates of oestrogen receptor concentrations that can be distinguished from zero, based on either the probability of detection, using practical laboratory data, or criteria derived from computer simulation studies. The data generated are of interest in relation to the establishment of the hormone sensitivity of tumours with low cytosol receptor levels and may be useful in assessing the possibly improved procedures for receptor measurement using monoclonal antibodies to receptor protein.

Correspondence: D.W. Wilson.

Received 12 June 1984; accepted 18 July 1984.

### Materials and methods

### *Oestradiol-17$\beta$ receptor assay*

Breast tumour tissue was stored in liquid $N_2$ and assayed within 2–3 weeks after surgery. Tissue ($\sim$0.5 g) was cut into small pieces, pulverised into a fine powder in an all-glass homogeniser in 3 ml buffer (10 mM TRIS; 1 mM EDTA; 10% v/v glycerol; 5 mM dithiothreitol; pH = 7.4). The high speed supernatant (100 $\mu$l; 105,000 *g* for 60 min) was incubated for 16 h at 4°C with 100 $\mu$l of one of 10 concentrations of [$^3$H] oestradiol (Sp. act. $\sim$ 100 Ci mmol$^{-1}$), ranging from 0.2–5.0 nmol l$^{-1}$ buffer. Aliquots of these solutions were taken for counting and from a knowledge of the counting efficiency and the specific activity a "better" estimate of oestradiol mass added to the incubation medium was calculated. Similar incubations were established in the absence of cytosol to assess the inefficiency of the procedure for separating free from bound hormone, which used DCC (200 $\mu$l; 0.5% gelatin, 0.05% dextran; 0.5% charcoal in TRIS buffer; 30 min at 4°C). Parallel incubations contained 100-fold excess of diethylstilboestrol at each oestradiol concentration

© The Macmillan Press Ltd., 1984

to assess non-specific binding. This assay has been used for all the receptor analyses associated with the Tenovus-Nottingham breast studies (Nicholson *et al.,* 1981).

*Protein determination*

The BIORAD assay kit (BIORAD, Dachauer Strasse 364, München, FRG) based on the method of Bradford (1976) was used for the determination of protein in breast tumour cytosol with bovine serum albumin as the protein standard. The cytosol (100 $\mu$l) was diluted in 0.15 M NaCl (900 $\mu$l) and 100 $\mu$l of the final solution was taken for assay in duplicate. The standard curve was set up using duplicate standards of 0, 10, 20, 40, 60 and 80 $\mu g\,ml^{-1}$. A linear regression analysis of optical density versus concentration enabled confidence limits of the standard curve to be established.

*Statistical analysis*

The practical data provided the total count rate (T) in the incubation medium which is a measure relating to oestradiol concentration, the inefficiency (X) of charcoal adsorption of free steroid, and the observed non-specific (NSB) and specific (S) count rates bound to the receptor site. Equations were derived to obtain estimates of the following true parameters: non-specific (N) and specific (S′) count rates bound to receptor sites; the "available" hormone concentration, which is related to the count rate (T′) that is free to react with specific binding sites; and the ratio of specifically bound to free oestradiol (R) in the incubation. These equations are published elsewhere by Richards *et al.* (1983).

The corrected binding data were then used to elicit the binding site concentration (q), using least squares regression analysis based on the linear Scatchard and non-linear Mass Action models. The relative biases associated with these models were then assessed. The sensitivity of detecting receptor levels as being distinct from zero was also evaluated using these corrected binding data. The accuracy of the Scatchard and non-linear Mass Action models and the effect of practical inefficiencies on the magnitude of receptor site concentration estimates, were also assessed by computer simulation. Chosen values of receptor site concentration and equilibrium association constant for the incubation, together with selected values for the mean and standard deviation of X, N and S′; enabled 40 sets of "practical" data to be generated at each receptor level using a Monte Carlo analysis technique based on the general Law of Mass Action. These simulated data were then re-analysed by the Scatchard and non-linear Mass Action models. It is important to emphasize that both models are different mathematical forms of the same mass action model. Consequently, this report is concerned with the statistical implications associated with the estimation of receptor site concentration using both these forms of the Mass Action model which are independent of the way the data have been generated. A more detailed account of the computer simulation and analysis is published elesewhere by Richards *et al.* (1983).

### Results and discussion

*Comparison of methods for calculating receptor site concentration*

The two methods for calculating the receptor site concentration in primary breast tumour cytosol were compared (Figure 1) for 23 sets of binding data, chosen at random, derived from the assay technique described. To aid visual comparison, values for the receptor site concentration were

**Figure 1** Comparison of Scatchard (▲) and non-linear least squares analysis of the Mass Action Model (○) using corrected binding data. Data are ranked according to non-linear estimates and a curve is drawn to aid visual assessment.

ranked in ascending order of magnitude based on the non-linear least squares Mass Action model and an arbitrary curve drawn through the points for visual continuity. In nearly all cases (21/23), the non-linear analysis gave lower values than the Scatchard model. Clearly, a different figure based on ascending order of Scatchard values could be drawn but the conclusions of relative bias remain unchanged. The accuracy and precision of both models were investigated using Monte Carlo analysis. The non-linear Mass Action model was found to be superior (Table I).

As an example, the coefficient of variation of an assay result ($CV_{R/P}$) expressed in fmol mg$^{-1}$ protein ($R_{f/p}$) was determined using the propagation of errors method (Melissinos, 1966) such that

$$CV_{R/P}=100(\sqrt{\sigma_R^2+(R_{f/p}\cdot\sigma_p)^2}/P)/[R_{f/p}]$$

where the standard deviation of receptor and protein measurements are denoted by $\sigma_R$ and $\sigma_p$ respectively and the corresponding mean values for the receptor and protein concentrations are given by R and P. In this example, $R=8.7$, $P=0.242$, $\sigma_R=1.31$, $\sigma_P=0.0121$ which leads to an assay result of 36 fmol mg$^{-1}$

**Table I** Minimum detectable receptor site concentration (MDRC) and associated bias and precision for Scatchard (S) and Mass Action (MA) models (fmol 100 $\mu$l$^{-1}$ cytosol).

| *Parameter* | *Non-specific binding (N)* | | | | | | | | | | | |
|---|---|---|---|---|---|---|---|---|---|---|---|---|
| | *N*=5% | | | | | | *N*=10% | | | | | |
| | %$CV_{(S')}$ | | | | | | %$CV_{(S')}$ | | | | | |
| | *10* | | *20* | | *30* | | *10* | | *20* | | *30* | |
| | *MA* | *S* | *MA* | *S* | *MA* | *S* | *MA* | *S* | *MA* | *S* | *MA* | *S* |
| True MDRC | 1 | 5 | 2 | 14 | 4 | — | 5 | 9 | 4 | 18[a] | 8 | — |
| Calculated MDRC, q | 1.4 | 6.2 | 2.4 | 21.0 | 4.9 | — | 5.8 | 12.0 | 5.8 | 31.8 | 10.0 | — |
| SD(q) | 1.2 | 0.8 | 1.2 | 3.6 | 2.2 | — | 2.4 | 2.5 | 3.8 | 19.0 | 6.1 | — |
| %CV | 86 | 13 | 50 | 17 | 45 | — | 41 | 21 | 66 | 60 | 61 | — |
| %BIAS | 40 | 24 | 20 | 50 | 23 | — | 16 | 33 | 45 | 77[a] | 25 | — |

$K=0.5\times10^{10}$ LM$^{-1}$; X=1%; CV(X)=30%; CV(Non-specific binding)=10%.
[a]The closest solution but does not satisfy criterion for bias.

*Limits of detection*: Practical data

Using this Mass Action model, attention was focused on the statistical uncertainties associated with measuring low values of receptor site concentrations so that the probability of detecting receptor levels distinct from zero, could be established and possible misclassification of receptor status in tumours minimized. Analysis of samples, ($n=5$), chosen at low receptor site concentrations ranging from 8.7–14.6 fmol 100 $\mu$l$^{-1}$ cytosol and protein content ranging from 0.24–0.54 mg 100 $\mu$l$^{-1}$ cytosol, when expressed as fmol mg$^{-1}$ of protein, ranged from 23.2 to 36.0. The pooled variance of the receptor estimations was 2.37 (fmol mg$^{-1}$ protein)$^2$ and the estimated standard deviation for protein concentrations between 0.2 to 0.6 mg 100 $\mu$l$^{-1}$ cytosol was ~5% of the mean value, this being obtained from the pooled sample variance obtained from 12 samples of cytosol.

protein with a coefficient of variation ($CV_{R/P}$) of 16%. Under routine conditions, 5% of the samples have a protein concentration in the cytosol of <0.10 mg 100 $\mu$l$^{-1}$, therefore the protein content requires re-analysis at lower dilutions to increase precision of measurement otherwise the final result may be considerably biased and will substantially affect the uncertainties associated with the value of the receptor level when expressed in fmol mg$^{-1}$ protein. In any case, the distribution of standards in the assay militate against precise estimates of protein concentrations below 0.10 mg 100 $\mu$l$^{-1}$ and the standard practice is to re-assay the cytosol at a lower dilution. The minimum detectable receptor concentration, $\Delta R$ is defined as, $\Delta R=K^1\sqrt{\sigma_{R_0}^2+\sigma_{R_{AR}}^2}$ If the constant $K^1$, selected on the basis of a percentage point for a Normal distribution, is equal to $\sqrt{2}$ ("standard deviations") and the variance in detecting "zero" receptor concentration ($\sigma_{R_0}^2$) is equal to the variance at $\Delta R$,

($\sigma^2_{R_{\Delta R}}$), a questionable assumption, then $\Delta R = 2\sqrt{2.37} \cong 3$ fmol which can be detected with a probability of 97.5%. A receptor value of 3 fmol would have its 95% confidence bounded by 0 and 6.0 fmol, which is equivalent to 0 to 15 fmol mg$^{-1}$ protein, assuming the protein content is 0.4 mg 100 $\mu$l$^{-1}$ cytosol with a negligible error of estimation. Even at $\sigma_p = 0.04$, this would be equivalent to a receptor value within the 95% confidence boundaries of 0 and 16 fmol mg$^{-1}$ protein. Clearly, since $n = 5$ then the between sample estimate of variance is subject to error; if samples are available, $n > 8$ is a preferrable basis for calculating $\Delta R$. Even so, $\sigma_{R_{\Delta R}}$ has not been estimated from a large sample size and so Student's *t*-distribution rather than the normal distribution is now used to calculate the detection limit. In the case where the limit is based on 2 standard deviations, the corresponding Student's *t*-value for $n = 5$ is 2.78 for 4 degrees of freedom which effectively increases estimates of the detection limit still further.

*Limits of Detection*: Simulated data

Simulation studies were undertaken to compare the Scatchard and non-linear least squares models. These used "corrected" data at low levels of receptor concentration, for selected values of both nonspecific binding and inefficiency of separating free from bound oestradiol and their associated uncertainties, together with those for specifically bound ligand. Certain criteria were used in the simulation studies, which are in-line with analytical practice, to ascertain the "minimum" detectable concentrations of receptor site distinguishable from zero. These criteria were (a) that for the Scatchard plot, 5% or less of the 40 data sets produced negative receptor values and (b) for the non-linear least squares method, 5% or less of the data sets failed to provide a convergent solution in the calculation of receptor content. To test the separate effect of the inefficiency of separation, (X), and non-specific binding, (N), on the minimum detectable receptor site concentration, either X or N were set equal to zero together with their respective uncertainty and that of specific binding, as described elsewhere (Richards *et al.*, 1983). However, an evaluation of the separate effects of inefficiencies on the reliable estimation of uncertainties for both models does not reflect the practical situation where interactions of uncertainties in from different sources of inefficiencies in the analytical system are present. To overcome this deficiency, *not discussed by Richards et al.*, (*1983*), simulation studies of interacting sources of uncertainty for receptor levels [where the uncertainty in specific binding was 10%, 20% or 30%; non-specific binding was either 5% or 10% with coefficients of variation of 10%; the inefficiency of separating free from bound hormone was 1% with a CV of 30% (conditions often encountered in practice)] gave data summarized in Table I. The parameters referred to in this table require explanation. The true minimum detectable receptor site concentration (MDRC) refers to the minimum integer value of receptor site concentration used to simulate binding data which on subsequent analysis provided the minimum calculated receptor site concentration which satisfied criteria for the limit of detection. As can be seen this limit is different for each method. The difference between the true and calculated values of MDRC, expressed as a percentage of the true value, provide an estimate of bias in numerical accuracy. The data in Table I indicate the advantage of the Mass Action (MA) model in detecting lower concentrations of receptor site concentration. Estimates of imprecision are best interpreted from the standard deviation (s.d.) since, by definition, the coefficient of variation (CV) approaches infinity as the value of q converges towards zero. For the purposes of further comparison, the MA model has been evaluated under the conditions of MDRC that can be achieved using the Scatchard (S) model. Thus comparable MA values for the true MDRC (fmol 100 $\mu$l$^{-1}$ cytosol) calculated MDRC (q), s.d.(q) and % bias under the same conditions as the Scatchard model (represented in Table I by columns 3, 5, 9 and 11) are 5.0, 5.2, 1.2, 23%, 4%; 14.0, 14.4, 4.5, 31.3%, 2.9%; 9.0, 9.0, 2.4, 27%, 0%; and 18.0, 19.8, 6.4, 32.3%, 10%, respectively. Although the imprecision of the Scatchard appears marginally better under conditions of low uncertainties in the various parameters, under conditions of high uncertainties the situation is reversed and under all conditions so far tested, the numerical accuracy of the MA model is superior. It was observed that when the level of uncertainty in specific binding was raised from 10% to 20% or 30%, greater difficulty was met when attempting to obtain satisfactory solutions for both the Scatchard plot and the non-linear squares analysis. Although it is impracticable to demonstrate all the interactions of different sources and magnitudes of methodological errors on imprecision, bias and detection limits for the receptor assay, nevertheless data may be generated by computer simulation which clearly show the limitations of the Scatchard and Mass Action models. Figure 2 illustrates the superiority of the Mass Action model with respect to bias and detection limits for a non-specific binding level of 5% (CV = 30%) for coefficients of variation in specific binding of 5%, 10% or 20%.

**Figure 2** Comparison of calculated values of the mean receptor site concentration obtained from 40 sets of simulated binding data at each true q level using the Scatchard (●), S, and non-linear Mass Action (□), MA, models. Data are for X = 1%, CV(X) = 30%; N = 5%, CV(N) = 30%; and the CV of specific binding is (a) 5%, (b) 10% and (c) 20%. The figures in parentheses refer to the number of positive q values obtained from 40 simulations when they are less than 38. The straight lines represent the condition of zero bias and MDRC is the minimum detectable receptor site concentration.

The value of the inefficiency of separating free from bound hormone is 1% (CV = 30%). The simulation data in Table I and those in Figure 2 are reproducible although some variation in the detection limits is to be expected since different sequences of random numbers are generated for each run. To avoid ambiguity it is emphasized that the coefficients of variation of X and N are expressed as a percentage of the percentage parameter mean. The computer program devised by Richards *et al.* (1983) may also be used to optimize the analytical procedure in terms of the distribution of the various concentrations of labelled oestradiol used in the assay and their order of replication. This could be an important feature of the program for use in a routine assay laboratory.

To illustrate the importance of sources of uncertainty on the "analytical cut-off point", e.g. 5 fmol 100 $\mu l^{-1}$, representative sets of 40 receptor site values at two levels of non-specific binding *viz.* 5% and 10%, with coefficients of variation of 10% or 20% are shown in Figure 3. The increased bias and imprecision of the Scatchard model are evident and would be even more exaggerated when receptor levels were expressed in fmol $mg^{-1}$ protein, assuming an average protein content of 0.4 mg 100 $\mu l^{-1}$ cytosol. At N = 10% (CV = 20%) only 25% of the generated receptor values are positive using the Scatchard model compared with 95% calculable values for the Mass Action model.

It must be emphasized that this report is intended to illustrate, by practical experiment and computer simulation, those factors which affect the minimum detectable receptor concentration, estimates of the precision of receptor measurements and their numerical bias. Clearly, the model used in the analysis is only meant to be an approximation since levels of non-specific binding, expressed as a percentage of each oestradiol concentration often depart from linearity; such deviations cannot readily be accommodated in a model that can have general applicability in the field. Factors associated

**Figure 3** Comparison of receptor site concentrations obtained from 40 sets of simulated binding data using Scatchard (-----) and Mass Action (——) models. The true equilibrium association constant and receptor site concentration are $0.5 \times 10^{10}\,LM^{-1}$ and 5 fmol $100\,\mu l^{-1}$ cytosol respectively. The coefficient of variation (CV) in specific binding is 10%, the inefficiency of separation is 1% (CV=30%) and the non-specific binding is (a) 5% (CV=10%), (b) 5% (CV=20%), (c) 10% (CV=10%) and (d) 10% (CV=20%). The mean value (and s.d.) are given for each data set in fmol $100\,\mu l^{-1}$ cytosol. The breaks in the Mass Action profile in (d) represent incalculable results; in the case of the Scratchard the co-ordinates are presented as solid circles.

with uncertainties arising from dissociation of the bound complex, as discussed in a report by Wilson *et al.* (1971), have not been incorporated in this model. Although this is a relatively minor problem for the oestradiol receptor assay it may be important in other binding systems. The mathematical model in terms of the response variable, R, is not claimed to be the "best", neither may a parametric model be the most robust method of analysis. What is clear is that the Scatchard model is not appropriate at low levels of receptor concentration, particularly in the presence of analytical "noise". Finally this report concerns, *inter alia,* analytical cut-off levels for receptor positivity in breast tumour cytosols. The degree of cellularity (McGuire *et al.,* 1977), and the existence of clones of ER-positive and ER-negative cells within the tumour, together with the presence of "non-functional" or type II receptors in the cytosol, may also influence the quantitative determination of the receptor status of "tumour" tissue.

In conclusion, a computer simulation technique has been described which allows a proper assessment of the uncertainties associated with the DCC method of assaying cytosolic oestradiol-17$\beta$ receptor proteins in human breast cancer tissue. It provides guide-lines for acceptable levels of inefficiencies in the analytical system, together with their uncertainties, as well as giving a more realistic assessment of the minimum detectable receptor concentration.

The generous financial support of the Tenovus Organisation is gratefully acknowledged. Mr Barrie Francis provided technical assistance and the British Quality Assessment Group for oestrogen receptors provided additional stimulus, particularly Dr Diana Barnes and Dr R. Leake.

### References

BRADFORD, M.M. (1976). A rapid and sensitive method for the quantitation of Microgram quantities of protein utilising the principle of protein-dye binding (see also, The Bio-Rad Laboratories Instruction Booklet, 1981). *Anal. Biochem.*, **72,** 248.

CHAMNESS, G.C. & McGUIRE, W.L. (1975). Scatchard plots: Common errors in correction and interpretation. *Steroids*, **26,** 538.

Consensus Meeting on Steroid Receptors in Breast Cancer, (1980) N.I.H., Bethesda, U.S.A. *Cancer*, **46,** 2759.

HAYBITTLE, J.L., BLAMEY, R.W., ELSTON, C.W. & 5 others (1982). A prognostic index in primary breast cancer. *Br. J. Cancer*, **45,** 361.

JENSEN, E.V., SMITH, S. & DE SOMBRE, E.R. (1976). Hormone dependency in breast cancer. *J. Steroid Biochem.*, **7,** 911.

MELISSINOS, A.C. (1966). In: *Experiments in Modern Physics*, New York, Academic Press.

McGUIRE, W.L., CARBONE, P.P., VOLLMER, E.P. (1975) (eds). In: *Estrogen Receptors in Human Breast Cancer*. New York, Raven Press.

McGUIRE, W.L., ZAVA, D. HORWITZ, K.B., CHAMNESS, G.C. (1978). Hormones, receptors and breast cancer. In: (Eds Griffiths *et al.*) *Tumour Markers* Cardiff, Alpha Omega p. 153.

McGUIRE, W.L., HOROWITZ, K.B., PEARSON, O.H. & SEGALOFF, A. (1977). Current status of estrogen and progesterone receptors in breast cancer. *Cancer*, **39,** 2934.

NICHOLSON, R.I., CAMPBELL, F.C., BLAMEY, R.W., ELSTON, C.W., GEORGE, D & GRIFFITHS, K. (1981). Steroid receptors in early breast cancer: Value in prognosis. *J. Steroid Biochem.*, **15,** 193.

RICHARDS, G., WILSON, D.W. & GRIFFITHS, K. (1983). Computer-aided assessment of receptor status in human breast cancer. *Comput Biomed. Res.*, **16,** 483.

WILSON, D.W., SARFATY, G., CLARRIS, B., DOUGLAS, M. & CRAWSHAW, K. (1971). The prediction of standard curves and errors for the assay of estradiol by competitive protein binding. *Steroids*, **18,** 77.


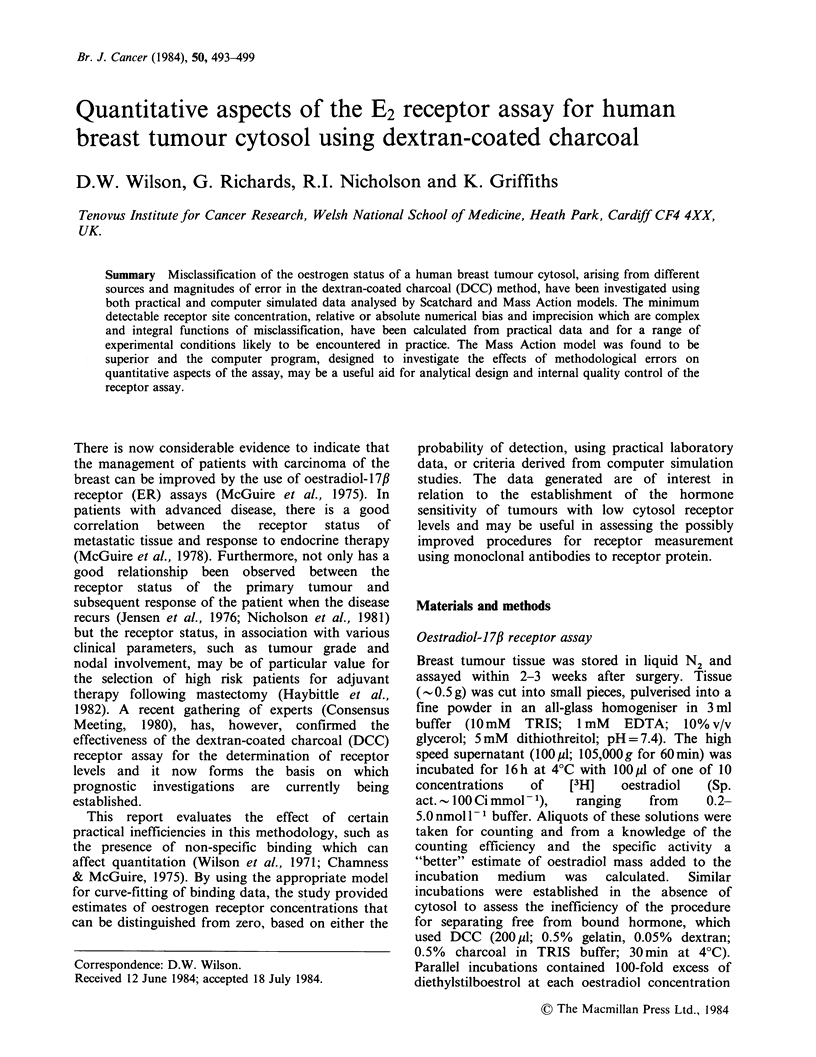

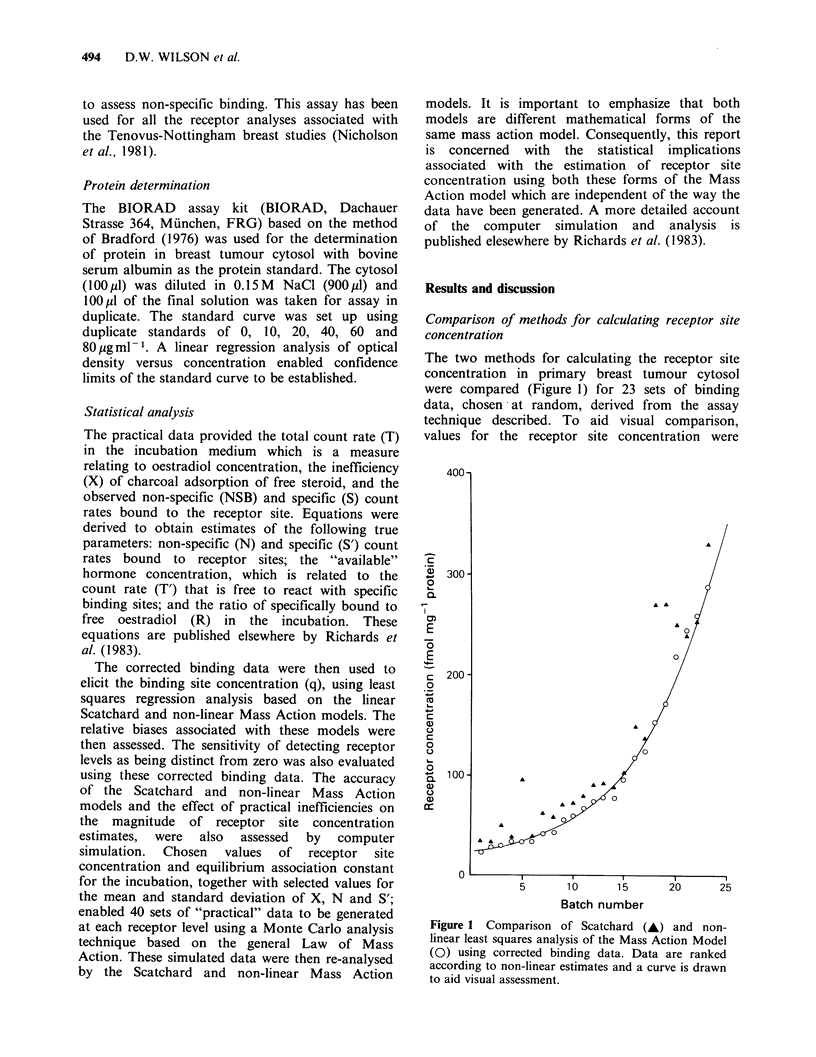

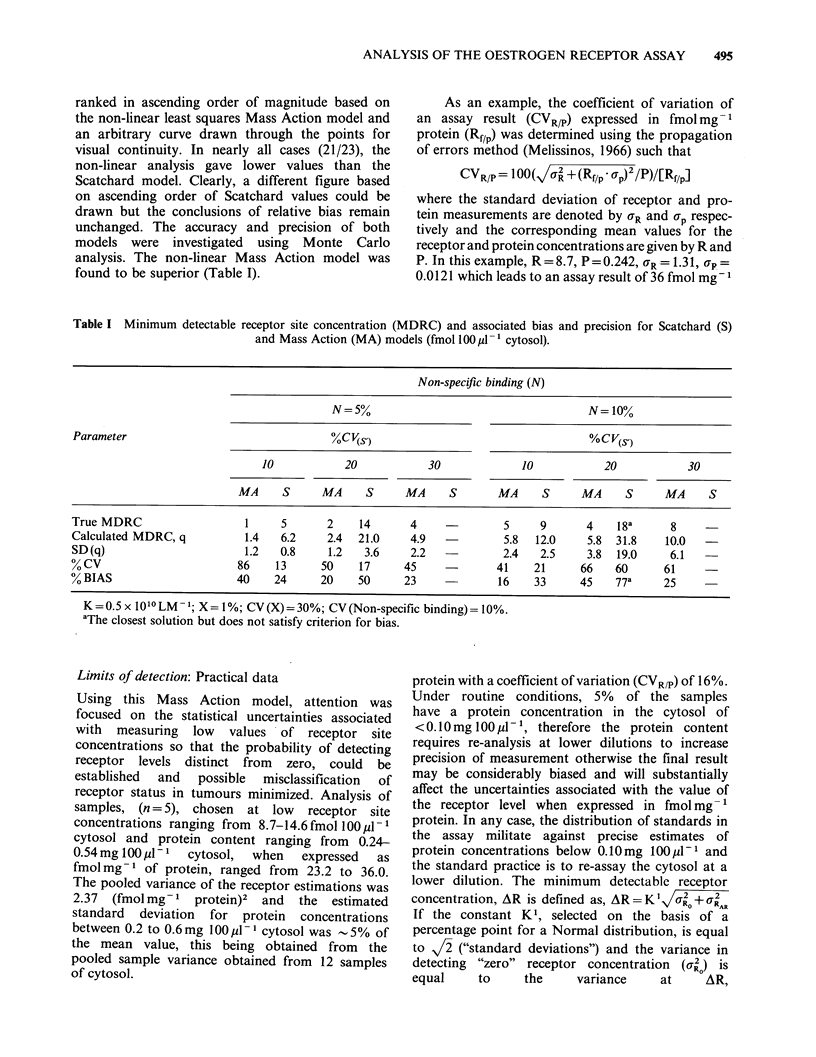

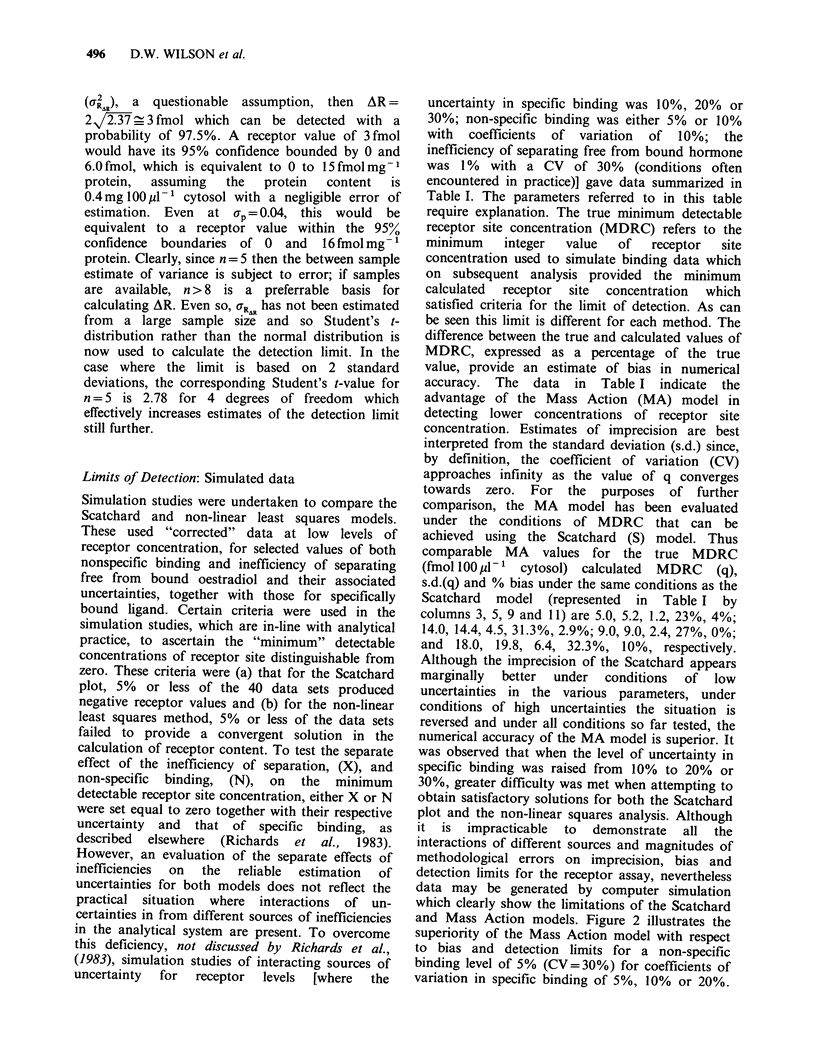

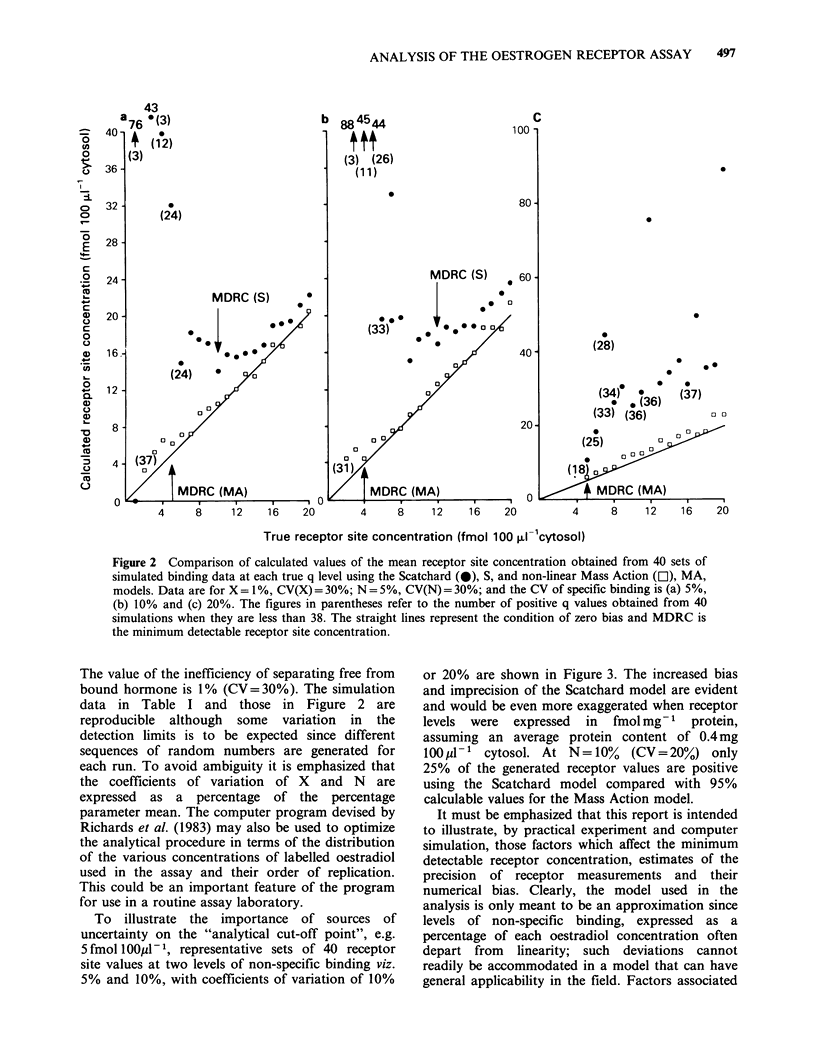

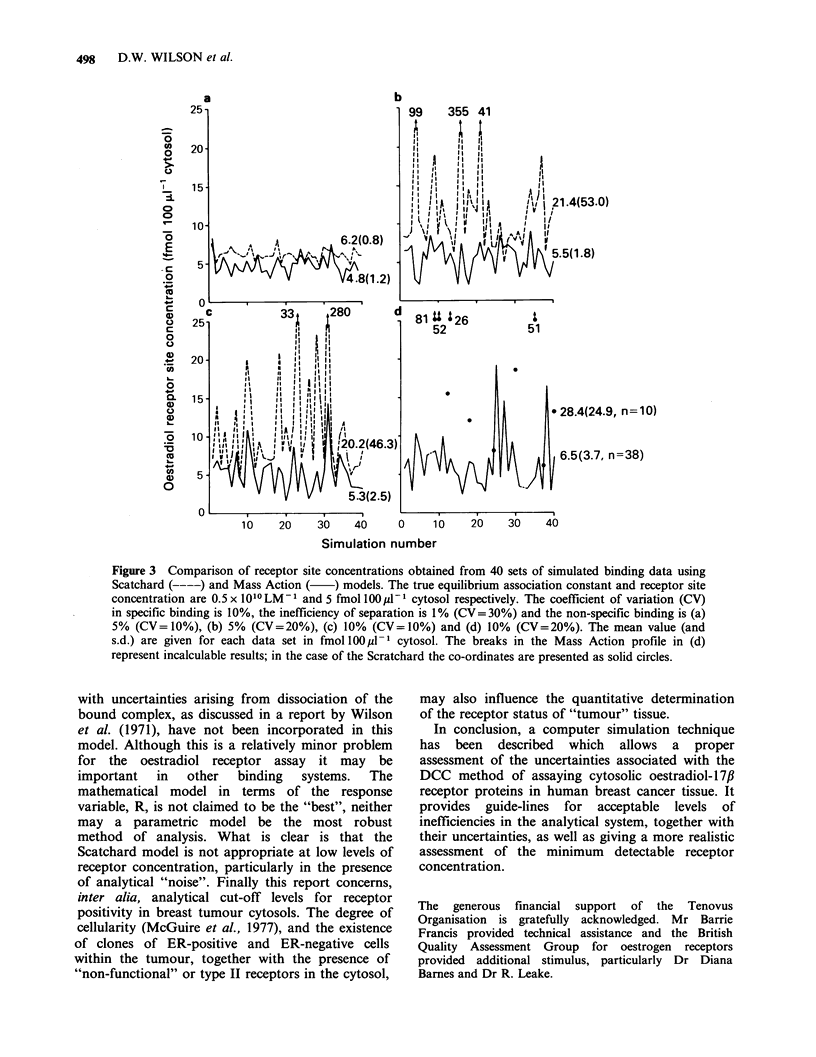

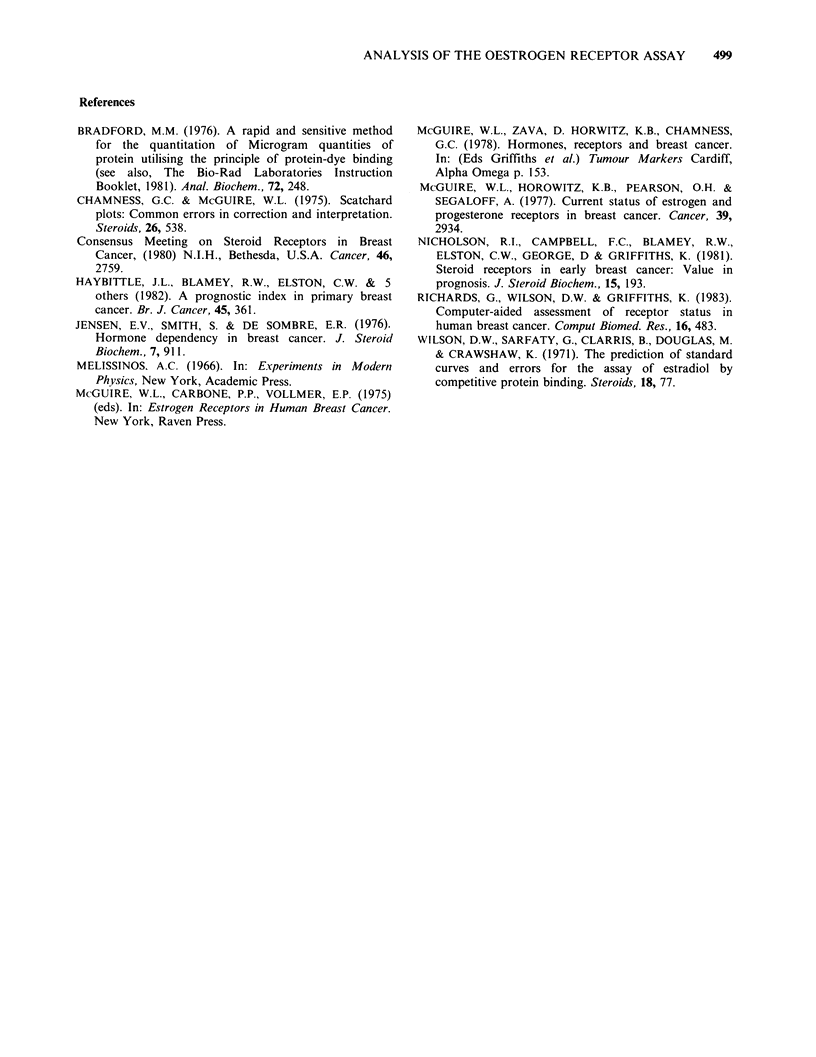


## References

[OCR_00732] Bradford M. M. (1976). A rapid and sensitive method for the quantitation of microgram quantities of protein utilizing the principle of protein-dye binding.. Anal Biochem.

[OCR_00739] Chamness G. C., McGuire W. L. (1975). Scatchard plots: common errors in correction and interpretation.. Steroids.

[OCR_00749] Haybittle J. L., Blamey R. W., Elston C. W., Johnson J., Doyle P. J., Campbell F. C., Nicholson R. I., Griffiths K. (1982). A prognostic index in primary breast cancer.. Br J Cancer.

[OCR_00754] Jensen E. V., Smith S., DeSombre E. R. (1976). Hormone dependency in breast cancer.. J Steroid Biochem.

[OCR_00774] McGuire W. L., Horwitz K. B., Pearson O. H., Segaloff A. (1977). Current status of estrogen and progesterone receptors in breast cancer.. Cancer.

[OCR_00780] Nicholson R. I., Campbell F. C., Blamey R. W., Elston C. W., George D., Griffiths K. (1981). Steroid receptors in early breast cancer: value in prognosis.. J Steroid Biochem.

[OCR_00786] Richards G., Wilson D. W., Griffiths K. (1983). Computer-aided assessment of receptor status in human breast cancer.. Comput Biomed Res.

[OCR_00791] Wilson D., Sarfaty G., Clarris B., Douglas M., Crawshaw K. (1971). The prediction of standard curves and errors for the assay of estradiol by competitive protein binding.. Steroids.

